# Bounds for phylogenetic network space metrics

**DOI:** 10.1007/s00285-017-1171-0

**Published:** 2017-08-23

**Authors:** Andrew Francis, Katharina T. Huber, Vincent Moulton, Taoyang Wu

**Affiliations:** 1 Centre for Research in Mathematics, Western Sydney University, Penrith, Australia; 2 School of Computing Sciences, University of East Anglia, Norwich, NR4 7TJ UK

**Keywords:** Phylogenetic networks, Spaces of phylogenetic networks, Phylogenetic network metrics, Nearest-neighbor interchange (NNI), Diameter, 05C90, 92D15

## Abstract

Phylogenetic networks are a generalization of phylogenetic trees that allow for representation of reticulate evolution. Recently, a space of unrooted phylogenetic networks was introduced, where such a network is a connected graph in which every vertex has degree 1 or 3 and whose leaf-set is a fixed set *X* of taxa. This space, denoted $\mathcal {N}(X)$, is defined in terms of two operations on networks—the nearest neighbor interchange and triangle operations—which can be used to transform any network with leaf set *X* into any other network with that leaf set. In particular, it gives rise to a metric *d* on ${\mathcal {N}}(X)$ which is given by the smallest number of operations required to transform one network in ${\mathcal {N}}(X)$ into another in ${\mathcal {N}}(X)$. The metric generalizes the well-known NNI-metric on phylogenetic trees which has been intensively studied in the literature. In this paper, we derive a bound for the metric *d* as well as a related metric $d_{N\!N\!I}$ which arises when restricting *d* to the subset of $\mathcal {N}(X)$ consisting of all networks with $2(|X|-1+i)$ vertices, $i \ge 1$. We also introduce two new metrics on networks—the SPR and TBR metrics—which generalize the metrics on phylogenetic trees with the same name and give bounds for these new metrics. We expect our results to eventually have applications to the development and understanding of network search algorithms.

## Introduction

**Fig. 1:**
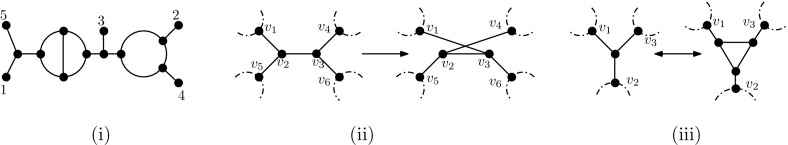
(i) Example of a phylogenetic network on the set $X=\{1,2,3,4,5\}$. This network is in tier 3, because it has $n=14$ vertices and $\ell =5$ leaves, and $14=2(5+3-1)$. It has two blobs. (ii) An NNI operation on adjacent degree three vertices, changing a path $v_1, v_2,v_3,v_4$ to $v_1,v_3,v_2,v_4$. (iii) The triangle operation that shifts between tiers ${\mathcal {N}}_i(x)$ and ${\mathcal {N}}_{i+1}(X)$, $i\ge 0$

Phylogenetic networks are a generalization of phylogenetic trees that are used to represent either non-tree-like evolutionary histories arising in organisms such as plants and bacteria, or uncertainty in evolutionary histories (Huson et al. 2010; Steel 2016). Here we are interested in *unrooted* binary phylogenetic networks on a finite set *X* of taxa, or *networks* for short. These are connected graphs in which every vertex has degree 1 or 3 and whose leaf-set is *X* (Gambette et al. 2012). An example of such a network is presented in Fig. 1(i). Note that if a network is a tree (i.e. it has no cycles), then it is also known as a *phylogenetic tree*. Networks can be generated from biological data using software such as T-REX (Makarenkov 2001) and have been used, for example, to study the origin of genomes in eukaryotes (Rivera and Lake 2004).

Recently, it has been shown that it is possible to transform any network on a set *X* into any other network on the same set using a finite sequence of two types of operations (Huber et al. 2016b). These operations are pictured in Fig. 1(ii) and (iii), and are called *nearest neighbor interchange* (NNI) and *triangle* operations, respectively. Note that the NNI operation generalizes the operation with the same name which is used to compare phylogenetic trees (Robinson 1971). In light of this result, as observed in Huber et al. (2016b), a space ${{\mathcal {N}}}(X)$ of phylogenetic networks on *X* may be defined as follows. It is the graph with vertex set consisting of all networks on *X*, and edges corresponding to pairs of networks which differ by either one NNI operation or one triangle operation. Since we can transform any network in ${\mathcal {N}}(X)$ into any other network in ${\mathcal {N}}(X)$ using a finite sequence of NNI and triangle operations, it follows that the space ${\mathcal {N}}(X)$ is connected.

The space ${{\mathcal {N}}}(X)$ generalizes a discretized version of tree-space (Billera et al. 2001), the graph with vertex set consisting of all phylogenetic trees on *X* with edges corresponding to pairs of trees which differ by one NNI operation. Indeed, it actually contains tree-space (on *X*) as a subspace as we shall now explain. For $i \ge 0$, we let ${\mathcal {N}}_i(X)$ denote the set of all *tier i* networks on *X*, that is, all networks on *X* with $2(|X|-1+i)$ vertices. A tier 3 example is shown in Fig. 1(i). Clearly the space ${\mathcal {N}}(X)$ is the disjoint union of the ${\mathcal {N}}_i(X)$ taken over $i \ge 0$. Moreover, ${{\mathcal {N}}}_0(X)$ is precisely the set of phylogenetic trees on *X*, and ${{\mathcal {N}}}_1(X)$ is the set of unicyclic networks on *X*  (see, e.g. Semple and Steel 2006). Each ${\mathcal {N}}_i(X)$ is a connected subgraph of ${\mathcal {N}}(X)$, where the edges correspond only to NNI operations (Huber et al. 2016b), so that tree-space is a subspace of ${\mathcal {N}}(X)$.

Tree-space is equipped with the *NNI metric* $d_{N\!N\!I}$, which for any two trees *T* and $T'$ contained in it is defined to be the minimum number of NNI operations required to transform *T* into $T'$. The NNI metric has been intensively studied in the literature  (see e.g. DasGupta et al. 1997), and its properties have important consequences for tree search algorithms. One such property is the diameter of tree space, where the *diameter* $\Delta (D)$ of a metric *D* on a set *Y* is its maximum value taken over all pairs of elements in *Y*. In Li et al. (1996) it is shown that, for $\ell =|X|\ge 3$, the diameter of $d_{N\!N\!I}$ satisfies

$$\begin{aligned} (\ell -4)/2 \log [(2\sqrt{2}/3e)(\ell -2)] \le \Delta (d_{N\!N\!I}) \le \ell \log (\ell ) +O(\ell ). \end{aligned}$$

The second bound improved on an $O(\ell ^2)$ upper bound given by Robinson (1971).

Network spaces are equipped with metrics which naturally generalize the NNI-metric on trees. In particular, for $N,N' \in {\mathcal {N}}_i(X)$ (or, more generally, $N,N' \in {\mathcal {N}}(X)$), we define the distance $d_{N\!N\!I}(N,N')$ (the distance $d_{{\mathcal {N}}(X)}(N,N')$) to be the minimal number of NNI operations (respectively, NNI and triangle operations) to transform *N* into $N'$. In this paper, we focus on giving bounds on the diameter of $d_{N\!N\!I}$ of ${\mathcal {N}}_i(X)$, and upper bounds for $d_{{\mathcal {N}}(X)}(N,N')$ for any $N,N' \in {\mathcal {N}}(X)$. Note that $d_{N\!N\!I}$ is bounded on ${{\mathcal {N}}}_i(X)$ (since $|{\mathcal {N}}_i(X)|$ is finite), whereas $d_{{\mathcal {N}}(X)}$ can become arbitrarily large on ${{\mathcal {N}}}(X)$. Hence it only makes sense to consider diameter bounds for the metric $d_{N\!N\!I}$ on ${{\mathcal {N}}}_i(X)$. As with tree-space, we expect that our results could eventually prove useful for network construction algorithms.

We now summarize the contents of this paper. After presenting some preliminaries in the next section, in Sect. 3 we begin by introducing a family of phylogenetic networks that we call “echidna” networks. We then exploit properties of these networks in Sect. 4, together with results on graph grammars in Sleator et al. (1992), to give a lower bound for the diameter of the metric $d_{N\!N\!I}$ on ${{\mathcal {N}}}_i(X)$ (see Theorem 2). An upper bound for the same diameter is then derived in Sect. 5 (see Theorem 3). To derive this bound, we exploit properties of Hamiltonian paths in the graph that arises from a network by removing its leaves and their adjacent edges. Using our upper bound on $d_{N\!N\!I}$, we also derive an upper bound for $d_{{\mathcal {N}}(X)}(N,N')$ for any $N,N' \in {\mathcal {N}}(X)$ (see Corollary 4).

In Sect. 6, we define SPR and TBR operations on networks. These operations generalize the NNI operation, as well as the well-known subtree prune and regraft (SPR) and tree bisection and reconnection (TBR) operations on trees (cf. Allen and Steel 2001). The SPR and TBR operations allow parts of a network to be chopped off and reconnected somewhere onto the resulting network, in contrast to the NNI and triangle operations which are local in nature. In Sect. 7, we derive bounds for the diameter of the SPR and TBR metrics on the set on ${{\mathcal {N}}}_i(X)$. We conclude in Sect. 8 with a discussion of some possible future directions.

## Preliminaries

For us, graphs contain no parallel edges (edges with the same pair of end vertices), and no loops (edges with one vertex as both end vertices). This means that edges are uniquely determined by a pair of vertices $\{v,w\}$ with $v\ne w$.

Suppose throughout that *X* is a finite set with $|X|\ge 3$. A *phylogenetic network* on leaf-set *X* (or a network (on *X*), for short) is a connected graph in which every vertex has degree 3 or degree 1, and in which the vertices of degree 1 are labelled by the elements of *X* (e.g. Fig. 1(i)). This means that a phylogenetic network is essentially a cubic graph (a graph in which every vertex has degree 3) with leaves attached. It also means that phylogenetic networks for us are *unrooted*, so that edges have no implicit direction.

Write *V*(*N*) for the set of vertices in *N* and *E*(*N*) for the set of edges of *N*. We will reserve *n* for the number of vertices in the network, $n:=|V(N)|$.

The concept of the *tier* of a network on *X* will be important for this paper, and has been defined in Sect. 1 (following Huber et al. 2016b). It is also known as the *reticulation number* of a network, because it is the number of edges one must remove from it for it to become a phylogenetic tree on *X* (Lemma 3).

A *cut-edge*, or *bridge*, of a network is an edge whose removal disconnects the graph. A cut-edge is *trivial* if one of the connected components induced by the cut-edge is a vertex and *non-trivial* otherwise. A *simple* network is one whose cut-edges are all trivial (so note, for instance, that trees on more than two leaves are *not* simple networks). A *blob* is a maximal subgraph of a network that has no cut-edge (i.e. a “biconnected component”, also known as a “block”), and that is not a vertex (Gambette et al. 2012).

There are several numbers associated with a network that will be widely used in this paper. The first, *n*, has already been mentioned: $n=|V(N)|$. Others are the size of the leaf-set, $\ell :=|X|$, and the tier of the network, which is a non-negative integer and which we will usually denote *i*. These three variables are related by the following equation, as stated in the Introduction:

$$\begin{aligned} n=2(\ell +i-1). \end{aligned}$$

In this paper we will consider networks that we call *pseudo-Hamiltonian*: networks that contain a cycle that passes through every non-leaf vertex. Note that every pseudo-Hamiltonian network is simple, but not vice versa. One can construct simple graphs that are not pseudo-Hamiltonian, by for instance taking a cubic graph that is not Hamiltonian, and adding some leaves to it.

The nearest-neighbour interchange (NNI) is a local operation, initially defined for phylogenetic trees, that is important for moving around tree-space in search algorithms. Such algorithms are vital for estimating phylogenetic trees using likelihood or parsimony methods. The NNI operation has also been defined as follows for phylogenetic networks (Huber et al. 2016b), since it is in a sense an operation on a pair of adjacent degree 3 vertices in a graph (see Fig. 1(ii)).

### Definition 1

(*NNI*) Let $v_1,v_2,v_3,v_4$ be a path in a network in which neither $\{v_1,v_3\}$ nor $\{v_2,v_4\}$ is an edge. An NNI operation on this path replaces it with the path $v_1,v_3,v_2,v_4$.

This replacement of a path has the effect of retaining the central edge $\{v_2,v_3\}$, while replacing edge $\{v_1,v_2\}$ with the new edge $\{v_1,v_3\}$ and edge $\{v_3,v_4\}$ with the new edge $\{v_2,v_4\}$.

We now briefly digress beyond a fixed tier and consider the wider network space ${\mathcal {N}}(X)$. The *triangle operation* introduced in Huber et al. (2016b), allows movement between tiers by inserting a 3-cycle at any degree-3 vertex (“blow-up”), or collapsing a 3-cycle into a degree-3 vertex (“collapse”). See Fig. 1(iii).

### Proposition 1

(Huber et al. 2016b) For each $i\ge 0$, ${\mathcal {N}}_i(X)$ is connected by NNI operations. Moreover, the space of networks ${\mathcal {N}}(X)$ is connected by NNI operations together with triangle operations.

Because the space ${\mathcal {N}}(X)$ is connected, the distance $d_{{\mathcal {N}}(X)}$ is well-defined, and indeed is a metric (as is the NNI distance on tier *i* networks) (Huber et al. 2016b, Theorem 5). As it turns out, a canonical extension of the notion of the subtree prune and regraft (SPR) and tree-bisect and regraft (TBR) operations for trees to networks (see Definitions 2 and 3 for precise details) allows us to establish the companion result for Proposition 1.

### Corollary 1

For each $i\ge 0$, ${\mathcal {N}}_i(X)$ is connected by SPR operations, and by TBR operations. Moreover, the space of networks ${\mathcal {N}}(X)$ is connected by SPR operations together with triangle operations, and by TBR operations together with triangle operations.

### Proof

Each NNI operation is an SPR operation, and hence also a TBR operation (this is easy to check and is noted in Lemma 7), so the result follows immediately from Proposition 1. $\square$

Finally for this preliminary section, we reiterate that this paper is focussed on movements within a single tier. The remarks about wider movement around the space ${\mathcal {N}}(X)$ in Proposition 1 and Corollary 1 are included for context.

Write $S_k$ for the symmetric group on the set $\{1,\dots ,k\}$, for $k\ge 1$. For the sake of extremal cases, we set $S_0$ to be trivial group consisting of the empty map from $\emptyset$ to itself. Similarly, we adopt the convention that $0!=1$ and $\log _a(0)=0$ for all $a>0$.

## Echidna graphs

The first main result of this paper, provided in Sect. 4, is a lower bound on the diameter of the space ${\mathcal {N}}_i(X)$ of tier *i* phylogenetic networks under NNI operations. To obtain this bound, we will need a lower bound on the number of phylogenetic networks in tier *i*. That lower bound is established in this section (Corollary 2), by counting the number of distinct networks in a subset of ${\mathcal {N}}_i(X)$. This subset is the set of *echidna* graphs^1^, which we will define shortly. Echidna graphs are useful for this purpose because they can be counted through a bijection with a set of sequences ${\mathcal {S}}(p,q)$, defined as follows.

For integers $p\ge 1$ and $q\ge 0$, define ${\mathcal {S}}(p,q)$ to be the set of sequences of length $p+q$ whose entries are the symbols $\{a_1,\dots ,a_p\}$ and *q* copies of 0, and that begin and end with $a_1$ and $a_p$ respectively. Denote the *k*th entry of a sequence $S\in {\mathcal {S}}(p,q)$ by *S*[*k*]. The number of such sequences is $|{\mathcal {S}}(p,q)|=\frac{(p+q-2)!}{q!}$.

For $\ell \ge 3$ and $i\ge 1$, we use a sequence $S\in {\mathcal {S}}(\ell ,i-1)$ and a permutation $\pi \in S_{i-1}$ (if $i>1$), to define a tier *i* network $G(S,\pi )$ with $\ell$ leaves labelled $\{1,\dots ,\ell \}$ as follows.

**Fig. 2:**
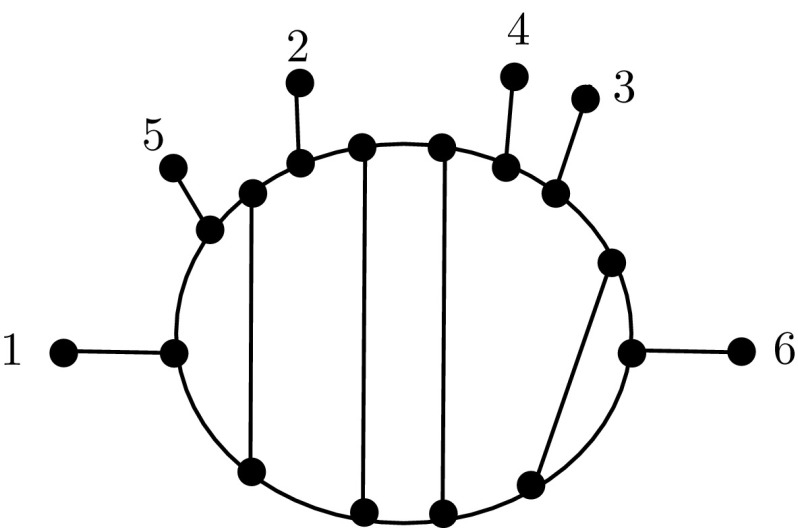
Example of a phylogenetic network $G(S,\pi _0)$ in the echidna family with $S=(a_1,a_5,0,a_2,0,0,a_4,a_3,0,a_6)$

Draw a circle and create $\ell +i-1$ degree 2 vertices labelled clockwise by the sequence elements *S*[*k*], for $1\le k\le \ell +i-1$, to obtain a cycle *C* with vertices $S[1],\dots ,S[\ell +i-1]$. Each vertex is thus labelled $a_j$ for some $j=1,\dots ,\ell$ or 0. To each of the $\ell$ vertices for which $S[k]=a_j\ne 0$, attach a leaf with label *j*. Next, subdivide the edge $\{S[1], S[\ell +i-1]\}$ by $i-1$ degree 2 vertices reading anticlockwise from *S*[1] to $S[\ell +i-1]$. Referring to the resulting graph also as *C*, draw $i-1$ *chords*, that is, edges from the degree 2 vertices along the top of *C* (those labelled 0) to the degree 2 vertices along the bottom of *C* according to the permutation $\pi$ (using implied numbering from their positions in the sequence). Denote this graph $G(S,\pi )$. An example with $\pi _0=id$ is shown in Fig. 2.

We call graphs constructed in this way *echidna graphs*, and for a given number of leaves $\ell$ and $i\ge 1$, denote the set of such graphs by

$$\begin{aligned} \mathcal {G}(\ell ,i-1):=\{G(S,\pi )\mid S\in {\mathcal {S}}(\ell ,i-1),\pi \in S_{i-1}\}. \end{aligned}$$

Note that elements of $\mathcal {G}(\ell ,i-1)$ are tier *i* phylogenetic networks, and are also *pseudo-Hamiltonian graphs*, as defined in Sect. 2.

In what follows, we will mostly restrict our attention to echidna graphs in which the permutation in $S_{i-1}$ is the identity map $\pi _0$. We will show that different sequences in ${\mathcal {S}}(\ell ,i-1)$ generate non-isomorphic graphs. We begin by noting some properties of echidna graphs generated from different sequences.

### Lemma 1

Write $G=G(S,\pi _0)$ and $G'=G(S',\pi _0)$ in $\mathcal {G}(\ell ,i-1)$, for $\ell \ge 3$ and $i \ge 1$. Suppose $S\ne S'$ and let *k* be the first position at which they differ, that is, $k\in \{2,\ldots ,\ell +i-2\}$ is such that $S[k]\ne S'[k]$ and $S[j]=S'[j]$, for all $1\le j\le k-1$. Suppose, without loss of generality, that $S[k]\ne 0$ (noting that at least one of *S*[*k*] and $S'[k]$ must be non-zero), so that $S[k]=a_\alpha$ for some $\alpha =2,\dots ,\ell -1$. In addition, assume that $d_G(1,\alpha )= d_{G'}(1,\alpha )$. Then either

(A) $S[k-1]=S'[k-1]=a_\beta$ for some $\beta =1,\dots ,\ell$. That is, the entry before $a_\alpha$ in *S* also corresponds to a leaf (namely $\beta$); or
(B) $S[k-1]=S'[k-1]=0$ is the last zero entry in *S* (and therefore $S'$), and $a_\alpha$ is the second last entry in $S'$. That is, $S'[\ell +i-2]=S[k]$.

### Proof

Firstly, we can rule out the case that $a_\alpha$ is the first entry in *S* after $a_1$, namely the case $k=2$, as follows. If $S[2]=a_\alpha$ then $d_G(1,\alpha )=3$, and so $d_{G'}(1,\alpha )=3$ by the assumption of the lemma. But by construction of the echidna graphs, the only way two leaves can be 3 apart is if their corresponding terms are consecutive in the graph’s defining sequence (excluding the case the two leaves are labelled 1 and $\ell$), and this forces $a_\alpha$ to also be the second entry of $S'$, a contradiction (since *S* and $S'$ differ at the *k*th position).

Now suppose $k>2$ and consider minimal paths from 1 to $\alpha$ in *G* and $G'$. Suppose that (A) does not hold, that is, that $S[k-1]=S'[k-1]=0$. We need to show that $S[k-1]$ is the last zero entry in *S*.

To this end, let *x* be the vertex that is adjacent to $S[k-1]$ that is not equal to its other neighbors $S[k-2]$ and *S*[*k*]. In addition, let *c* be the chord containing *x* and $S[k-1]$. Since the removal of *x* and $S[k-1]$ disconnects *G* into a graph in which 1 and $\alpha$ are in different components, it follows that a minimal path from 1 to $\alpha$ must go through $S[k-1]$ or *x*. Therefore, either

1 $$\begin{aligned} d_G(1,\alpha )&=d_G(1,S[k-1])+d_G(S[k-1],\alpha )\nonumber \\&=d_G(1,S[k-1])+2, \end{aligned}$$

(since the distance from $S[k-1]$ to $\alpha$ is 2), or

2 $$\begin{aligned} d_G(1,\alpha )&=d_G(1,x)+d_G(x,\alpha ) \nonumber \\&=d_G(1,x)+3, \end{aligned}$$

(since the distance from *x* to $\alpha$ is 3, noting that $\alpha \ne \ell$). This can be seen because there certainly *is* a path of length 3 from *x* to $\alpha$ (namely $x,S[k-1], a_{\alpha }, \alpha$), and in general any path from *x* to $\alpha$ must contain at least four vertices, namely *x*, $S[k\pm 1]$, *S*[*k*], and $\alpha$.

**Fig. 3:**
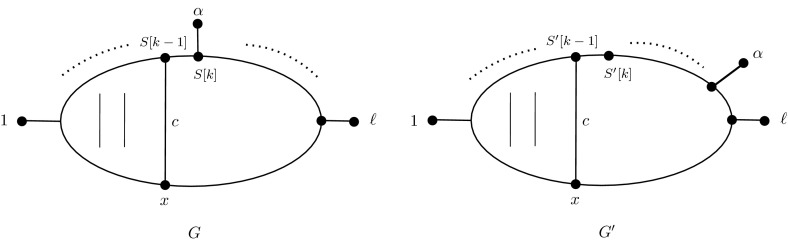
The situation of case (B) in Lemma 1. All chords that are different from *c* (indicated by *two vertical parallel lines*) are to the left of chord *c* in both graphs

By definition of *k* and the fact that *x* and $S[k-1]$ are in the chord *c*, it follows that $d_G(1,S[k-1])=d_{G'}(1,S'[k-1])$ and $d_G(1,x)=d_{G'}(1,x)$, labelling *x* and *c* in $G'$ as in *G* (see Fig. 3). Indeed, in either graph, paths from 1 to $S[k-1]$ must go through $S[k-2]$ or *x*, both of which are distance 1 from $S[k-1]$ (with an equivalent statement for $S'$).

The only way $S'[k-1]$ could be distance 2 from $\alpha$ in $G'$ is if $a_\alpha =S'[k]$ or $S'[k-2]$, both of which are ruled out by assumptions. This eliminates the possibility that a minimal path from 1 to $\alpha$ in $G'$ may go through $S'[k-1]$. Since paths from 1 to $\alpha$ in $G'$ must, as in *G*, go through either $S'[k-1]$ or *x*, we have from (2) that $d_{G'}(x,\alpha )=3$.

Any path of length 3 from *x* to $\alpha$ in $G'$ that goes up the chord *c* would similarly force $a_\alpha =S'[k]$ or $S'[k-2]$, both not possible. So the path of length 3 from *x* to $\alpha$ in $G'$ does not contain *c*. It also cannot go towards the leaf labelled 1 since that is further from $\alpha$. Therefore it goes towards the leaf labelled $\ell$, from *x*.

If there was another chord in $G'$ coming after *c*, then any path from *x* to $\alpha$ going up that chord would have distance at least 4: the path along the bottom from *x* to the new chord; the chord itself; the path along the top from the top of the chord to $a_\alpha$; and the edge to the leaf $\alpha$ itself.

Therefore, there is no chord coming after *c* in $G'$, and so we are in situation (B): *c* is the last chord, in the $(k-1)$th position (so that $S'[k-1]$ is the last zero in $S'$, and hence also in *S*), and the position of $\alpha$ in $G'$ must be adjacent to that of the final leaf, $\ell$. This situation is illustrated in Fig. 3. $\square$

We are now able to prove our main result about echidna graphs: that distinct (non-isomorphic) echidna graphs are generated by distinct sequences.

### Theorem 1

Write $G=G(S,\pi _0)$ and $G'=G(S',\pi _0)$ in $\mathcal {G}(\ell ,i-1)$ for $\ell \ge 3$ and $i\ge 1$. If $S\ne S'$ in ${\mathcal {S}}(\ell , i-1)$, then $G\not \cong G'$.

### Proof

Suppose *k* is the first position for which $S[k]\ne S'[k]$. Then this position is non-zero in at least one of *S* and $S'$, and so without loss of generality suppose that $S[k]=a_\alpha$ with $\alpha >1$.

If $d_G(1,\alpha )\ne d_{G'}(1,\alpha )$, then $G\not \cong G'$, and we are done. So suppose that $d_G(1,\alpha )= d_{G'}(1,\alpha )$.

By Lemma 1, either (A) or (B) holds.

If (A), then there exists some leaf $\beta \ne 1$ such that $S[k-1]=S'[k-1]=a_\beta$. But then $d_G(\alpha ,\beta )=3$, while $d_{G'}(\alpha ,\beta )>3$, by definition of *k* and since distances between leaves can only be 3 if their corresponding terms are adjacent in the sequence.

If (B), note that $a_\alpha$ in *G* is not adjacent to $a_\ell$, since if it were then $S=S'$ ($a_\alpha$ is the first point at which they differ). However $a_\alpha$ in $G'$ *is* adjacent to $a_\ell$. This implies that $d_{G'}(\alpha ,\ell )=3<d_G(\alpha ,\ell )$, and so the graphs are not isomorphic. $\square$

### Corollary 2

The number of tier *i* phylogenetic networks on *X*, with $|X|=\ell \ge 3$ and $i\ge 1$, is

$$\begin{aligned} |{\mathcal {N}}_i(X)|\ge \frac{(\ell +i-3)!}{(i-1)!}. \end{aligned}$$

Moreover, when $i=1$ we have

$$\begin{aligned} |{\mathcal {N}}_1(X)|\ge (\ell -2)!2^{\ell -3}. \end{aligned}$$

### Proof

The number of distinct echidna graphs with $\pi =\pi _0$ is at least the number of sequences in ${\mathcal {S}}(\ell ,i-1)$, namely $\frac{(\ell +i-3)!}{(i-1)!}$, and the set of such echidna graphs is a subset of the set of tier *i* phylogenetic networks.

For $i=1$, we have

$$\begin{aligned} |{\mathcal {N}}_1(X)|=(\ell -1)!2^{\ell -2}-\frac{(2\ell -2)!}{(\ell -1)!2^{\ell -1}} \ge (\ell -2)!2^{\ell -3}, \end{aligned}$$

where the first equality follows from Semple and Steel (2006, Theorem 3) and the second inequality follows from

$$\begin{aligned} \frac{(2\ell -2)!}{(\ell -1)!2^{\ell -1}}\frac{1}{(\ell -1)!2^{\ell -2}} \le \frac{2\ell -3}{2\ell -2} \quad \text{ and }\quad \frac{(\ell -2)!2^{\ell -3}}{(\ell -1)!2^{\ell -2}} \le \frac{1}{2\ell -2}. \end{aligned}$$

$$\square $$

Note, this result also holds for $i=0$ because there are $(2\ell -5)!!$ trees and $(2\ell -5)!!\ge (\ell -3)!$.

### Remark 1

It would be interesting to see whether the term $(i-1)!$ can be removed from the denominator of the general bound in Corollary 2 for $i>1$. One way to achieve this might be to count echidna networks for general $\pi \in S_{i-1}$, but it seems that this is not trivial.

## A lower bound on the NNI diameter

In this section we provide a lower bound on the maximum distance between two tier *i* phylogenetic networks under NNI operations (Theorem 2). Our strategy follows that of Li et al. (1996), who construct bounds for a similar NNI diameter on tree-space. The strategy involves first bounding the number of networks in a ball of given radius around a network (Proposition 2), then using upper and lower bounds on the size of a factorial (Lemma 2). For the former of these, we follow Li et al. (1996) in using the concept of a “graph grammar”, from Sleator et al. (1992).

### Proposition 2

The number of networks in ${\mathcal {N}}_i(X)$ reachable in *m* or fewer NNI operations from any given network in ${\mathcal {N}}_i(X)$ is at most $6^{2(\ell +i-1)+10m}$.

### Proof

**Fig. 4:**
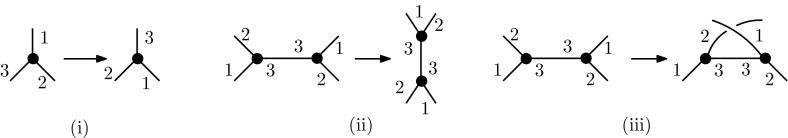
The graph grammar of productions that implement NNI operations. The labels are on half edges

Define a graph grammar by the three “productions” shown in Fig. 4 (to use the language of  Sleator et al. (1992)). There is one “triplet” production (see Fig. 4(i)) and two “quartet” productions (see Fig. 4 (ii) and (iii)) .

For each vertex of degree 3, label half-edges in *N* arbitrarily by 1,2,3. Any NNI operation on a quartet in *N* involves at most five of the productions in Fig. 4: up to two rotations of labels for each vertex, performed by the triplet production, to align the labels with the quartet productions, plus one of the quartet productions. Thus, a sequence of *m* NNI operations becomes a sequence of at most 5*m* productions in the graph grammar. Now, applying Theorem 2.3 of Sleator et al. (1992), the number of networks in ${\mathcal {N}}_i(X)$ reachable in *m* or fewer steps from any network in ${\mathcal {N}}_i(X)$ is $(c+1)^{n+5rm}$, where $c=5$ (the number of vertices on the left side of the grammar), $r=2$ (the largest number of vertices on the right side of any one production), and $n=2(\ell +i-1)$ (the number of vertices in the network). This completes the proof. $\square$

Note that the leaf labels in a phylogenetic network are “tags” in the sense of Sleator et al. (1992), and by Sleator et al. (1992, Remark 3.4), this does not change the formula in  Sleator et al. (1992, Theorem 2.3) for (leaf-labelled) phylogenetic networks.

We will exploit Stirling’s well-known formula giving bounds on *m*!, stated below.

### Lemma 2

(Stirling’s formula) For $m\ge 1$,

$$\begin{aligned} \sqrt{2\pi } \frac{m^{m+\frac{1}{2}}}{e^m}\le m!\le \sqrt{2\pi } \frac{m^{m+\frac{1}{2}}}{e^{m-1}}. \end{aligned}$$

Using Stirling’s formula, we have

### Corollary 3

For $|X|=\ell >3$, we have

$$\begin{aligned} |{\mathcal {N}}_1(X)|\ge \frac{(\ell -2)^{\ell -\frac{3}{2}}}{e^{\ell -1}}. \end{aligned}$$

### Proof

This follows from Corollary 2 and the fact that $2^{\ell -3}\sqrt{2\pi }>e$ holds for all integer $\ell$ greater than 3.$\square$

### Theorem 2

The diameter $\Delta _i$ of the set of tier *i* phylogenetic networks, $i\ge 1$, is bounded below by a term of order $n\log n$. More precisely

$$\begin{aligned} \Delta _i\ge \frac{1}{20}\left[ (n-3)\log _6\left( \frac{n}{2}-2\right) -(2i-1)\log _6(i-1)-(n-2i)\log _6e-2n \right] . \end{aligned}$$

Here we use $\log _6(0)=0$.

### Proof

Assume first that $i>1$. By Proposition 2, the number of networks reachable in $\Delta _i$ operations is at most $6^{n+10\Delta _i}$. But since this is the diameter, this is all networks. Thus from Corollary 2, we have

3 $$\begin{aligned} 6^{n+10\Delta _i} \ge \frac{(\frac{n}{2}-2)!}{(i-1)!}. \end{aligned}$$

Using Lemma 2, with $m=\frac{n}{2}-2$ for the numerator and $m=i-1$ for the denominator of Eq. (3), this gives:

$$\begin{aligned} 6^{n+10\Delta _i}&\ge \left[ \sqrt{2\pi } \frac{(\frac{n}{2}-2)^{\frac{n}{2}-\frac{3}{2}}}{e^{\frac{n}{2}-2}} \right] \times \left[ \frac{e^{i-2}}{(i-1)^{i-\frac{1}{2}}} \right] \\&= \frac{\sqrt{2\pi }(\frac{n}{2}-2)^{\frac{n}{2}-\frac{3}{2}}}{e^{\frac{n}{2}-i}(i-1)^{i-\frac{1}{2}}} \, . \end{aligned}$$

Taking logs base 6 and reorganising gives

$$\begin{aligned} \Delta _i&\ge \frac{1}{10}\left[ \log _6{\sqrt{2\pi }}+\frac{1}{2}(n-3)\log _6\left( \frac{n}{2}-2\right) -\frac{1}{2}(n-2i)\log _6e\right. \\&\quad \left. -\frac{1}{2}(2i-1)\log _6(i-1)-n \right] \\&\ge \frac{1}{20}\left[ (n-3)\log _6\left( \frac{n}{2}-2\right) -(2i-1)\log _6(i-1)-(n-2i)\log _6e-2n \right] , \end{aligned}$$

as required. This completes the proof for $i>1$. When $i=1$, the theorem holds for $\ell =3$ in view of $n=6$, and the case $\ell >3$ can be established in a similar way to that of $i>1$ by using Corollary 3. $\square$

## An upper bound on the NNI diameter

In this section we establish an upper bound on the NNI diameter of the space of phylogenetic networks, by providing a schematic NNI path between any two networks. The maximal length of this path is then an upper bound for the diameter of the space (Theorem 3).

The path we construct is as follows: first convert *N* into a simple network, and then into a pseudo-Hamiltonian network (defined in Sect. 2). Upper bounds for the number of steps in these conversions are given in Lemmas 4 and 5 respectively. We then show how to convert any pseudo-Hamiltonian network into any other in a bounded number of steps (Lemma 6).

Finally we remark in Corollary 4 that this result can be used to bound the distance between an arbitrary pair of networks in possibly different tiers.

We begin by deriving an upper bound on the number of non-trivial cut-edges for a network in tier *i*.

Given a connected graph *G* with vertex set *V* and edge set *E*, we consider $r(G) = |E|-|V|+1$. Note that *r*(*G*), known as the *cyclomatic number* of *G*, is clearly the number of edges that need to be removed from *G* in order to obtain a tree that is a spanning tree for *G*. For a network $N \in {\mathcal {N}}(X)$, *r*(*N*) is also known as the *reticulation number of N*.

### Lemma 3

Let $N \in {\mathcal {N}}(X)$ and $i\ge 0$. Then $N \in {\mathcal {N}}_i(X)$ if and only if $r(N)=i$.

### Proof

We show first that for any network $N\in {\mathcal {N}}_i(X)$ we have $|E(N)|=2\ell -3+3i$. Suppose $N \in {\mathcal {N}}_i(X)$. Then by Huber et al. (2016b, Theorem 3), we can obtain *N* by first taking some phylogenetic tree (i.e. a network in tier 0) which has $2\ell -3$ edges, then performing *i* triangle operations (which adds 3*i* edges) to obtain a network in ${\mathcal {N}}_i(X)$, and then performing some sequence of NNI operations to get *N* (which does not change the number of edges). Hence $|E(N)|=2\ell -3+3i$, as required.

Now, suppose that $N \in {\mathcal {N}}(X)$, with $N \in {\mathcal {N}}_i(X)$ for some $i\ge 0$, then $r(N) = |E(N)|-|V(N)|+1 = (2\ell -3+3i)- 2(\ell +i-1) + 1 = i$. Conversely, suppose $r(N)=i$. If $N \in {\mathcal {N}}_j(X)$ some $j\ge 0$, then $i=r(N)=|E(N)|-|V(N)|+1 = (2\ell -3+3j) - 2(\ell +j-1) + 1 = j$. $\square$

### Proposition 3

Let $N \in {\mathcal {N}}_i(X)$ for some $i\ge 0$, with *n* vertices. The number of non-trivial cut-edges in *N* is at most $\ell +i-3$.

### Proof

Without loss of generality, we may assume that $i\ge 1$ as otherwise *N* is a phylogenetic tree on *X* and that the result clearly holds. We consider the phylogenetic tree *T* on *X* that is obtained by shrinking each blob in *N* down to a vertex. Note that the number of non-trivial cut-edges in *N* is clearly at most the number of edges in *T* minus $\ell$.

Now, it follows by Huber et al. (2016b, Lemma 6), that by shrinking a blob *B* of *N* down to a vertex, we lose at least *r*(*B*) vertices. But *r*(*N*) is the sum of the values *r*(*B*) taken over all blobs *B* in *N*. Hence, since $r(N)=i$ by Lemma 3 and $|V(N)|=2(\ell +i-1)$, the tree *T* has at most $2\ell +i-2$ vertices, and so it has at most $2\ell +i-3$ edges. The proposition now follows immediately. $\square$

### Lemma 4

Suppose $N\in {\mathcal {N}}_i(X)$ and $i\ge 1$. We can convert *N* into a simple network by performing at most $\ell +i-3$ NNI operations on *N*.

### Proof

**Fig. 5:**
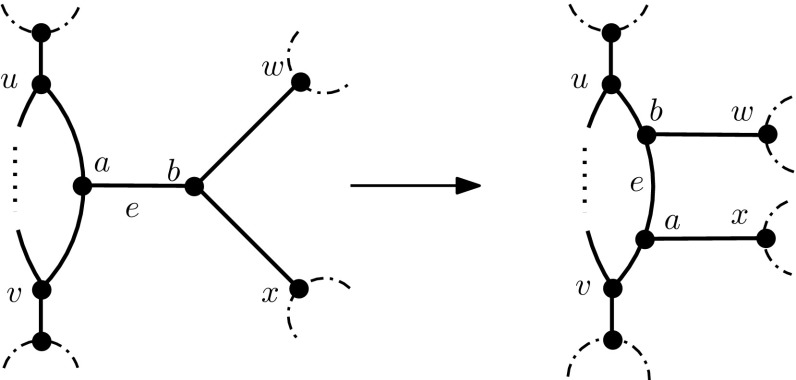
Example of an NNI operation on a cut-edge *e* that is incident with a vertex in a blob

Without loss of generality we may assume that *N* is not simple as otherwise the lemma clearly holds. Since $i>0$, *N* contains at least one blob. Let *e* be a non-trivial cut-edge of *N* and let $a,b\in V(N)$ such that $e=\{a,b\}$. Furthermore, let $u\in V(N)$ such that *u* is adjacent with *a* and let $w\in V(N)-\{a\}$ such that *w* is adjacent with *b*. Finally, let *C* denote the connected component of *N* containing *a*, obtained by deleting the edge *e*. For the convenience of the reader, we depict in Fig. 5 the case that *C* contains a cycle which shares a vertex with *e* and that the other vertex of *e* is not contained in a cycle of *N*.

Since *e* is a cut-edge of *N* we can perform an NNI operation on the path *u*, *a*, *b*, *w* to obtain a new network $N'$. Since *e* has been incorporated into *C* in $N'$ and no new cut-edge has been created by that operation, it follows that $N'$ has one cut-edge less than *N*. Consequently, by performing at most the number of non-trivial cut-edges NNI operations, we can convert N into a simple network.

The statement follows, by Proposition 3. $\square$

We now give an algorithm to convert *N* into a pseudo-Hamiltonian network.

### Lemma 5

Suppose $N\in {\mathcal {N}}_i(X)$ is simple, with $i\ge 1$. We can convert *N* into a pseudo-Hamiltonian network by performing at most *n* NNI operations on *N*.

### Proof

Suppose *N* is not pseudo-Hamiltonian. Since *N* is simple it must contain a blob *B*, and every cut-edge of *N* is trivial. Choose a maximal length cycle *C* in *B*.

Choose a non-leaf vertex *v* that is not in *C*, but is adjacent to a vertex $w\in V(C)$. Since *w* has degree 3 there must exist a vertex $w_1\in V(C)$ that is adjacent with *w*. Note that $w_1$ and *v* can not be adjacent, since if they were, they would be in a cycle that is longer than *C*, violating maximality (the path $w_1$ to *w* could be extended by going through *v*). Since *v* is not a leaf of *N*, we may choose another vertex $v_1\in V(N)-\{w\}$ that is adjacent to *v*. Again, since *w* has degree 3, and is contained in a cycle and adjacent to *v* (outside the cycle), the edge $\{w,v_1\}$ also is not contained in *N*. Hence, we may apply an NNI operation on the path $w_1,w,v,v_1$ to obtain a new network $N'$.

Note that $N'$ is still a simple network, but the cycle *C* has been extended to create a new cycle $C'$ with one more vertex, namely *v*. To see that $N'$ is still simple, let $v_2\in V(N)- \{w,v_1\}$ be the “other” vertex adjacent to *v* in *N*. Consider the edges $\{v,v_1\}$ and $\{v,v_2\}$ in *N*. Because *N* is simple, at most one of $v_1$ and $v_2$ is a leaf, and the other is (or both are) part of a path $w,v,v_i,\dots$ that begins and ends in a vertex in the cycle *C*. If one of $v_1,v_2$ was a leaf attached to *v* in *N*, it remains a leaf in $N'$, but is now attached to the cycle $C'$. For $v_i$ not a leaf, it is part of a path back to *C* in *N*, and remains part of a path back to $C'$ in $N'$ (a path that is now one vertex shorter).

It follows that a sequence of NNI operations can be performed, each increasing the length of a maximal length cycle in the network by one vertex. This process ends, since the number of vertices is finite, and it ends with a pseudo-Hamiltonian network. The number of NNI operations taken is at most the number of vertices in *N*, which completes the proof. $\square$

In our final lemma before the main theorem, we bound the distance between any two pseudo-Hamiltonian networks.

### Lemma 6

Any two pseudo-Hamiltonian networks in ${\mathcal {N}}_i(X)$, $i\ge 1$, are at most $$\left( {\begin{array}{c}\frac{n}{2}+i-1\\ 2\end{array}}\right) $$ NNI operations apart, where $n=|V(N)|$.

### Proof

Fix a pseudo-Hamiltonian cycle for each network. Both these cycles are of the same length, namely $n-\ell =\ell +2(i-1)$, and both have the same number, $\ell$, of vertices adjacent to leaves.

The leaves of each network are labelled 1 to $\ell$; number the adjacent vertex of each leaf by the same label. These vertices are on the pseudo-Hamiltonian cycle. Now, for each network, and for each non-leaf edge that is *not* on the pseudo-Hamiltonian cycle (that is, each *chord* ), number its end vertices by pairs $\{\ell +1,\ell +2\},\dots ,\{\ell +2i-3,\ell +2i-2\}$. This gives every vertex in each network a label ($\ell$ leaves, $\ell$ leaf-adjacent vertices, and $2(i-1)$ vertices contained in chords, for a total of $2(\ell +i-1)$).

For any two adjacent vertices $v_2,v_3$ on a pseudo-Hamiltonian cycle, performing an NNI operation on the length three sub-path $v_1,v_2,v_3,v_4$ has the effect of swapping the middle two adjacent vertices to give the sub-path $v_1,v_3,v_2,v_4$. Consequently, the arrangement of the vertices labelled $1,\dots ,\ell +2(i-1)$ on the pseudo-Hamiltonian cycles can be sorted between the two networks by NNI operations in the number of swaps of adjacent vertices in the cycle. This is bounded by the diameter of the symmetric group on $\ell +2(i-1)$, which is $$\left( {\begin{array}{c}\ell +2(i-1)\\ 2\end{array}}\right) =\left( {\begin{array}{c}n-\ell \\ 2\end{array}}\right) $$, as required, noting that $n-\ell =\frac{n}{2}+i-1$. $\square$

We can now give an upper bound for the diameter of ${\mathcal {N}}_i(X)$.

### Theorem 3

The diameter $\Delta _i$ of ${\mathcal {N}}_i(X)$, with $i\ge 1$, is bounded above by a term of order $n^2$. More precisely, we have

$$\begin{aligned} \Delta _i \le 3n+\left( {\begin{array}{c}\frac{n}{2}+i-1\\ 2\end{array}}\right) -2. \end{aligned}$$

### Proof

By Lemmas 5.2 and 5.3, any network in tier $i\ge 1$ can be transformed into a pseudo-Hamiltonian network in at most $\frac{n}{2}-1+n$ steps: $\frac{n}{2}-1=\ell +i-3$ to become simple and *n* to become pseudo-Hamiltonian. So given any two networks in ${\mathcal {N}}_i(X)$, they can be both made pseudo-Hamiltonian in a total of $3n-2$ steps, and, by Lemma 6, one can be transformed to the other in $$\left( {\begin{array}{c}\frac{n}{2}+i-1\\ 2\end{array}}\right) $$ steps. $\square$

Note that the same bound for $i=0$ follows immediately from Li et al. (1996) (where the bound is better than in this statement, being essentially $\ell \log \ell$).

We can use Theorem 3 to find an upper bound for the distance between *any* pair of networks in ${\mathcal {N}}(X)$, irrespective of tier.

### Corollary 4

Let $N,N'\in {\mathcal {N}}(X)$, with $N\in {\mathcal {N}}_i(X)$ and $N'\in {\mathcal {N}}_j(X)$. Suppose without loss of generality that $0\le i\le j$.

Then

$$\begin{aligned} d_{{\mathcal {N}}(X)}(N,N')\le 6\ell +7j-i-8+\left( {\begin{array}{c}\ell +2j-2\\ 2\end{array}}\right) . \end{aligned}$$

### Proof

By performing $j-i$ triangle operations starting with *N* we can create a network $N''$ in tier *j*. The distance from $N''$ to $N'$ is bounded above by the diameter bound $\Delta _j$ given in Theorem 3 (in which *n* is the number of vertices of a network in tier *j*, namely $n=2(\ell +j-1)$). Hence, the distance from *N* to $N'$ is at most $j-i + \Delta _j$, and

$$\begin{aligned} j-i + \Delta _j&\le j-i+3n+\left( {\begin{array}{c}\frac{n}{2}+j-1\\ 2\end{array}}\right) -2\\&=j-i+6(\ell +j-1)+\left( {\begin{array}{c}(\ell +j-1)+j-1\\ 2\end{array}}\right) -2\\&=6\ell +7j-i-8+\left( {\begin{array}{c}\ell +2j-2\\ 2\end{array}}\right) \end{aligned}$$

as required. $$\square $$

## SPR and TBR operations

**Fig. 6:**
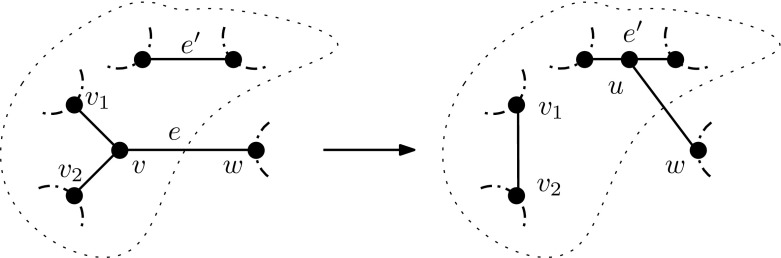
Example of an SPR operation. The operation is applied on *e*

In this section, we define “subtree prune and regraft” (SPR) and “tree bisection and regraft” (TBR) operations on network space ${\mathcal {N}}_i(X)$, $i\ge 1$. We extend the results of the previous sections on NNI operations to these operations on network space in Sect. 7. To state these definitions for some network $N\in {\mathcal {N}}(X)$, let $v,w\in V(N)$ such that *v* is of degree 3. Assume that $e=\{v,w\}$ is an edge in *N*, but that $\{v_1,v_2\}$ is not an edge in *N*, where $v_1,v_2$ are the vertices other than *w* incident to *v* in *N* (Fig. 6).

### Definition 2

(*SPR operation*) An *SPR operation* on *e* first removes *e* from *N* and then suppresses *v* (the degree of *v* is now 2). Next, it attaches a new edge $\{w,u\}$ to *w*, where *u* is a vertex subdividing an edge $e'$ of *N* not adjacent to *e*. In case *e* is a cut-edge of *N* then we also require that $e'$ is contained in the connected component not containing *w*.

### Definition 3

(*TBR operation*) Assume that *w* is such that the degree of *w* is also 3 and that $\{w_1,w_2\}$ is not an edge in *N* where $w_1,w_2\in V(N)-\{v\}$ are the other two vertices in *N* incident with *w*. A *TBR operation* on $e=\{v,w\}$ deletes the edge, suppressing the resulting degree 2 vertices *v* and *w*, and adds a new edge on *N* between a subdivision vertex of an edge $e_1$ and a subdivision vertex of a further edge $e_2$ of *N*. In case *e* is again a cut-edge of *N*, we also require that $e_1$ and $e_2$ are contained in distinct connected components.

Note that in Batagelj (1981) similar operations are defined on cubic graphs (see generating rules P1–P10).

It is straightforward to check that in network space each SPR operation is a TBR operation. Moreover, each NNI operation is also an SPR operation by noting that an NNI operation on a path $v_1,v_2,v_3,v_4$ results in a network that can be obtained from an SPR operation on $\{v_3,v_4\}$. So we state the following without proof.

### Lemma 7

$$N\!N\!I\subseteq S\!P\!R\subseteq T\!B\!R$$.

We will write $d_\Theta$ for the distance under the operation $\Theta$, for $\Theta \in \{N\!N\!I,S\!P\!R,T\!B\!R\}$. Note that these are distances *within* a tier, since each operation $\Theta$ is an operation that remains in a fixed tier. We have already noted in Sect. 2 that $d_{N\!N\!I}$ is a metric and, in view of the last lemma, it is straight-forward to check that the same also holds for $d_{S\!P\!R}$ and $d_{T\!B\!R}$.

In fact any TBR operation can be done by just two SPR operations, giving the following relationship among corresponding distances:

### Lemma 8

The SPR distance $d_{S\!P\!R}(N,N')\le 2d_{T\!B\!R}(N,N')$, the TBR distance, for networks $N,N'\in {\mathcal {N}}_i(X)$.

### Proof

Each TBR operation on an edge *e* of a network $N\in {\mathcal {N}}(X)$ can be performed by a pair of SPR operations where in the first SPR operation the role of *v* is played by one of the two vertices incident with *e* and, in the second, that role is played by the other vertex incident with *e*. $\square$

## Upper and lower bounds on the SPR and TBR diameters of ${\mathcal {N}}_i(X)$

We write ${\mathcal {N}}_i^\Theta (X)$ for the space of networks in tier *i* under the operation $\Theta \in \{N\!N\!I,S\!P\!R,T\!B\!R\}$, and write $\Delta _i^\Theta (n)$ for the diameter of ${\mathcal {N}}_i^\Theta (X)$ with $n=|X|$.

When $i=0$, ${\mathcal {N}}_0(X)$ is the set of phylogenetic trees on *X* and  Ding et al. (2011, Theorem 1.1) obtained the following bounds for $\Delta _0^{T\!B\!R}$ and $\Delta _0^{S\!P\!R}$:

$$\begin{aligned} \frac{n}{2}-\sqrt{2n} \le \Delta _0^{T\!B\!R} (n) \le \Delta _0^{S\!P\!R} (n) \le \frac{n-1}{2}-\frac{\sqrt{n-2}}{2\sqrt{2}} \end{aligned}$$

for $n\ge 6$. Therefore in this section we focus on the bounds for $i\ge 1$.

The number of SPR operations from any given network *N* in tier $i\ge 2$ can be given an upper bound as follows.

First, there is the number of edges one may choose for the operation. The number of edges is half the total degree, which is $3n-2\ell$ (each vertex has degree 3 except the leaves, which have degree 1). Note, $3n-2\ell =2(n+i-1)$, and so the number of edges is $n+i-1$.

Each edge *e* has two end vertices that may be chosen to be detached, and then one may regraft *e* on to any edge except *e* itself and the edges still incident to it: $n+i-4$ choices.

Thus there are at most

$$\begin{aligned} 2(n+i-1)(n+i-4) \end{aligned}$$

networks reachable from any network in ${\mathcal {N}}_i(X)$ by applying one SPR operation.

Setting $d=\Delta _i^{S\!P\!R}(n)$, following the previous logic of Sect. 4, we have that the number of networks in ${\mathcal {N}}_i(X)$ is at most $(2(n+i-1)(n+i-4))^d$, and so we have

$$\begin{aligned} (2(n+i-1)(n+i-4))^d\ge \frac{\sqrt{2\pi }(\frac{1}{2}n-2)^{\frac{1}{2}n-\frac{3}{2}}}{e^{\frac{1}{2}n-i}(i-1)^{i-\frac{1}{2}}} \end{aligned}$$

using the calculation in the proof of Theorem 2. Taking natural logs:

$$\begin{aligned} d\left[ \ln 2+\ln (n+i-1)+\ln (n+i-4)\right]\ge & {} \frac{1}{2}(n-3)\ln \left( \frac{n}{2}-2\right) \nonumber \\&-\frac{1}{2}(2i-1)\ln (i-1)-\frac{1}{2}(n-2i). \end{aligned}$$

Therefore,

$$\begin{aligned} \Delta _i^{S\!P\!R} (n)&\ge \frac{(n-3)\ln \left( \frac{n}{2}-2\right) -(2i-1)\ln (i-1)-(n-2i)}{2(\ln 2+\ln (n+i-1)+\ln (n+i-4))}\\&\ge \frac{(n-3)\ln \left( \frac{n}{2}-2\right) -(2i-1)\ln (i-1)-(n-2i)}{4\ln 2(n+i)} \, . \end{aligned}$$

This lower bound on the diameter $\Delta _i^{S\!P\!R}$ gives us one for the TBR diameter:

### Proposition 4

For $i\ge 1$ and $n\ge 6$, we have

$$\begin{aligned} \Delta _i^{T\!B\!R}(n) \ge \frac{1}{2}\Delta _i^{S\!P\!R}(n) \ge \frac{(n-3)\ln \left( \frac{n}{2}-2\right) -(2i-1)\ln (i-1)-(n-2i)}{8\ln 2(n+i)}. \end{aligned}$$

Here we use $\ln 0=0$.

### Proof

The first inequality follows from Lemma 8. The secondary inequality follows from the argument before the proposition for $i>1$. When $i=1$, it holds for $n=6$, and the case $n>6$ (and hence $\ell >3$) can be established in a similar way to that of $i>1$ by using Corollary 3. $\square$

To obtain upper bounds for $\Delta _i^{S\!P\!R}$ and $\Delta _i^{T\!B\!R}$, we can similarly follow our approach from the NNI case.

To move from a phylogenetic network $N\in {\mathcal {N}}_i(X)$ to another network $N'\in {\mathcal {N}}_i(X)$, first, convert the phylogenetic networks into pseudo-Hamiltonian forms, $N_1$ and $N_1'$. This takes at most 2*n* operations for each network, since that is how many NNI operations it takes (Lemmas 4 and 5). Combining Lemmas 6 and 7, at most $n^2$ SPR operations are needed to transform $N_1$ to $N_1'$.

This gives an upper bound for the SPR diameter of

$$\begin{aligned} \Delta _i^{S\!P\!R}(n) \le n^2+4n. \end{aligned}$$

Since each SPR operation is also a TBR operation (Lemma 7), the number of TBR operations between any two networks is at most the maximum number of SPR operations. That is, $d_{T\!B\!R}\le d_{S\!P\!R}$, which gives an upper bound on $\Delta _i^{T\!B\!R}$. These upper bounds are summarized as follows:

### Proposition 5

$$\begin{aligned} \Delta _i^{T\!B\!R}(n) \le \Delta _i^{S\!P\!R}(n) \le n^2+4n. \end{aligned}$$

Both upper bounds in Proposition 5 could be improved by an improvement on the upper bound for the number of SPR moves required to move between two pseudo-Hamiltonian networks. Whether that bound of $n^2$ can be improved is a question that may be of independent interest.

## Discussion

In this paper, we have presented upper and lower bounds for the diameter of the metric $d_{\Theta }$ on ${\mathcal {N}}^{\Theta }_i(X)$, $\Theta \in \{ N\!N\!I,S\!P\!R,T\!B\!R\}$. It would be interesting to know if these bounds can be improved upon and how close they are to being sharp. We suspect that the lower bound given in Theorem 2 could be improved by finding a larger lower bound for the number of networks in ${\mathcal {N}}_i(X)$ than the one given in Corollary 2, but have not been able to show this.

It would also be of interest to obtain a deeper understanding of the relationship between the structure of the space ${\mathcal {N}}(X)$ under $d_{\Theta }$ and the subspace obtained by restricting $d_{\Theta }$ to ${\mathcal {N}}_i^{\Theta }(X)$, $i\ge 0$. For example, it is clear that ${\mathcal {N}}_i^{\Theta }(X)$ is not an isometric subspace of ${\mathcal {N}}(X)$ under the metric $d_{\Theta }$ for $i \ge 1$, by virtue of the following example. Take a network *N* in tier *i*, and use the triangle operation to blow up a vertex *v*, giving a new network $N'$ in tier $i+1$. Now repeat this operation on *N* but this time use the triangle operation on a different vertex $w\ne v$, to get a different network $N''$ in tier $i+1$. The distance between $N'$ and $N''$ in ${\mathcal {N}}(X)$ is 2, through two judicious uses of the triangle operation. But the distance between them staying within tier $i+1$ is strictly greater than 2, regardless of which operation of NNI, SPR or TBR is used.

In this paper we have considered unrooted networks. However, it would be very interesting to see how our results could be extended to rooted networks. Some results concerning spaces of rooted networks are presented in Radice (2011) and in Nakhleh (2010). However, it is still necessary to define operation-based metrics on these spaces, and previous work on spaces of level-1 rooted networks in Huber et al. (2016a) suggests that this could be quite technical. Moreover, to find diameter bounds on the resulting space of rooted network metrics such as the one given in Theorem 2, it may be necessary either to introduce a new approach for dealing with graph grammars arising from directed graphs (which are not considered in Sleator et al. 1992), or to avoid this method of proof completely.

There are also some interesting computational questions concerning the metrics $d_{\Theta }$. For example, what is the computational complexity of computing $d_{\Theta }$? Note that the NNI, SPR and TBR distance are all NP-complete to compute (cf. DasGupta et al. 1997; Hickey et al. 2008; Allen and Steel 2001). In light of this fact, it is likely that the metric $d_{\Theta }$ is also NP-complete to compute. One way to show this could be to prove that ${\mathcal {N}}_0^{\Theta }(X)$ (i.e. tree-space) is an isometric subgraph of ${\mathcal {N}}^{\Theta }(X)$ under $d_{\Theta }$, which is a special case of the problem mentioned above.

In this paper we have considered discrete spaces of networks. However, it would be interesting to define and study continuous variants of these spaces. Continuous tree-spaces have been defined and studied by Billera et al. (2001), and arise since real-valued edge-lengths are often assigned to phylogenetic trees. How should we formally define continuous spaces of networks with edge-weights and metrics on these spaces, and what are their properties? Note that recently a definition for a continuous space of unrooted networks has been proposed in Devadoss and Petti (2016), and shown to have interesting geometric properties.

## Footnotes

1 Named for their resemblance to the Australian monotreme.
